# The Story of the Insane from Year to Year

**Published:** 1900-02-17

**Authors:** 


### THE STORY OF THE INSANE FROM YEAR TO YEAR.

Twelfth Series.

### THE ROYAL ASYLUM, MONTROSE.

During the year ending May 14th, 1899, the numbers in this asylum increased from 636 to 670. There were 149 first admissions, 40 readmissions, 68 recoveries, 33 discharged relieved, and 53 deaths. Calculated on the year's admissions the recoveries stand at 35·97 per cent., and the deaths calculated on the average number resident at 8·03 per cent. Of the deaths 24 were caused by cerebral and spinal diseases, 12 by consumption, and 1 by pneumonia. The usual table showing the causes of insanity in the admissions of the year is not given, nor could we find the statement of the average weekly maintenance rate. Referring to the 12 deaths from consumption Dr. Havelock says that during the year 14 patients were admitted suffering from this disease, and that as far as possible such cases are treated apart from the others, and spend as much time as possible in the open air. In this asylum the night nursing seems very efficiently carried out. There are 10 night attendants and nurses for 670 patients. There are 96 private patients in the asylum, and for these £4,321 was received. This would be about £45 a year each. It would be interesting to have a tabulated statement of these patients so that we might form some opinion of the amount of charitable work carried out. Some of these patients are placed in a block by themselves, and superior accommodation is provided for them. Another detached block for 60 patients is being erected at a cost of £5,500. If this block should be successful it is to be followed by others, and by these means the present overcrowding will be relieved. The committee "record their satisfaction with the services of the medical superintendent and his staff," and the commissioners say "the asylum was everywhere found in excellent order."

